# Lessons from early life: understanding development to expand stem cells and treat cancers

**DOI:** 10.1242/dev.201070

**Published:** 2022-10-11

**Authors:** Fiona M. Bain, James L. C. Che, Maria Jassinskaja, David G. Kent

**Affiliations:** Department of Biology, York Biomedical Research Institute, University of York, York, YO10 5DD, UK

**Keywords:** HSC, Haematopoiesis, Stem cells

## Abstract

Haematopoietic stem cell (HSC) self-renewal is a process that is essential for the development and homeostasis of the blood system. Self-renewal expansion divisions, which create two daughter HSCs from a single parent HSC, can be harnessed to create large numbers of HSCs for a wide range of cell and gene therapies, but the same process is also a driver of the abnormal expansion of HSCs in diseases such as cancer. Although HSCs are first produced during early embryonic development, the key stage and location where they undergo maximal expansion is in the foetal liver, making this tissue a rich source of data for deciphering the molecules driving HSC self-renewal. Another equally interesting stage occurs post-birth, several weeks after HSCs have migrated to the bone marrow, when HSCs undergo a developmental switch and adopt a more dormant state. Characterising these transition points during development is key, both for understanding the evolution of haematological malignancies and for developing methods to promote HSC expansion. In this Spotlight article, we provide an overview of some of the key insights that studying HSC development have brought to the fields of HSC expansion and translational medicine, many of which set the stage for the next big breakthroughs in the field.

## Teaser

**Summary:** This Spotlight summarises how our understanding of haematopoietic development *in vivo* can be applied to expand haematopoietic stem cells in vitro and aid our understanding of the development, and possible treatment, of blood cancers.

## Introduction

This is an exciting time for studying haematopoietic stem cells (HSCs), with new protocols for expanding mouse HSCs *in vitro* being translated to human HSCs and the molecular drivers of haematological malignancies being mapped to an unprecedented level. This ability to produce large numbers of HSCs has opened the door to a wide range of experimental assays previously considered impossible to perform on HSCs owing to the scarcity of this cell type. Moreover, experiments that have required herculean efforts and hundreds to thousands of mice can now be performed using only dozens. As such, the next decade promises to enable researchers to dissect in detail the molecular mechanisms governing HSC behaviour at the level of the epigenome, transcriptome, proteome and signallome (Bode et al., 2021).

Although this progress in expanding HSCs *in vitro* has been truly astounding, we sometimes forget to consider that the process of blood stem cell expansion still happens most efficiently during development and has been optimised evolutionarily. Specifically, the expansion of HSCs in the foetal liver – a hotspot for HSC self-renewal and expansion – and the subsequent switch of HSCs to a more dormant state in the juvenile bone marrow are incredibly interesting developmental stages from which to draw information.

This Spotlight article highlights how our knowledge of haematopoietic development *in vivo* underpins our understanding and utilisation of HSCs *in vitro* and in a clinical context, in particular with regard to HSC self-renewal, quiescence and expansion – processes that can be subverted to drive cancers or harnessed to provide new opportunities for therapeutic advances.

## Development of the haematopoietic system

Developmental haematopoiesis in mice occurs in separate waves, referred to as primitive and definitive (Dzierzak and Bigas, 2018; Ottersbach et al., 2010; Ivanovs et al., 2017). Primitive, HSC-independent haematopoiesis is first observed around embryonic day (E) 7.5 in mice (E17 in humans) in the yolk sac blood islands. In this wave, erythro-myeloid progenitors (EMPs) are formed in order to provide the early embryo with red blood cells and macrophages, the latter of which persist in adult life as tissue-resident immune cells (e.g. microglia, Langerhans cells and Kupffer cells) (Dzierzak and Medvinsky, 1995; Mirshekar-Syahkal et al., 2014). EMPs are now considered definitive embryonic progenitors, presenting with a marked difference in immunophenotype and function compared with primitive haematopoietic progenitor populations (Frame et al., 2016; McGrath et al., 2015). A second primitive wave produces some early myeloid and lymphoid cell types, including IL-7RA-expressing lymphomyeloid primed progenitors (LMPPs) (Böiers et al., 2013). However, because these primitive cells lack long-term self-renewal capacity, the primitive wave is considered transient (Orkin and Zon, 2008). Definitive haematopoiesis begins subsequently at E10.5 (E21 in human) in the aorta gonad mesonephros (AGM) region (Ivanovs et al., 2017), generating the first transplantable, definitive HSCs (Dzierzak, 2002). These rare, multipotent cells are produced from the haemogenic endothelium via endothelial-to-haematopoietic transition (EHT), a series of controlled morphological changes occurring in the vascular wall of the main embryonic arteries. This process is triggered by the onset of circulation and has been experimentally shown to be dependent on *Runx1* (Chen et al., 2009; Swiers et al., 2010). The onset of circulation and the associated production of nitric oxide (NO) from closely associated vascular endothelial cells have been shown to be essential regulators of haematopoiesis, with their influence beginning as early as the initiation of HSC formation in the AGM (North et al., 2009; Adamo et al., 2009). Exemplifying this, HSCs in *Ncx*^−/−^ (also known as *Slc8a1*) mice, which completely lack a circulation, are unable to develop past the pro-HSC stage, remaining functionally impaired with a dysregulated metabolism and an inability to activate the *Runx1* pathway (Azzoni et al., 2021). Alternative sites of definitive haematopoiesis have also been described, including the placenta (Mikkola et al., 2005; Gekas et al., 2010); however, some controversy around the magnitude of this contribution remains (Huang et al., 2007).

The extravasation of HSCs into the circulation allows them to migrate to future haematopoietic sites (Horton et al., 2021). Following a brief HSC expansion period in the placenta (Gekas et al., 2005), the foetal liver (FL) becomes the main site of definitive haematopoiesis by E11 and E12, with the most rapid phase of HSC expansion occurring at approximately E14.5, until HSCs migrate to the spleen (Christensen et al., 2004) and the bone marrow (BM) just before birth. Splenic haematopoiesis remains active until approximately 2 weeks after birth; from then on, and throughout adulthood, the BM is the primary site of HSCs and haematopoiesis (Rowe et al., 2016b) with HSCs largely acting as a dormant reservoir of non-dividing cells.

## The HSC foetal-to-adult switch

**Fig. 1.**
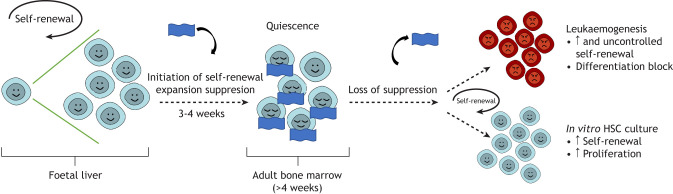
**Regulation of HSC self-renewal during life.** During development, particularly in the foetal liver, HSC self-renewal expansion divisions occur in order to produce HSCs to sustain lifelong haematopoiesis. Postnatally, at approximately 3-4 weeks of age in mice, this self-renewal expansion is suppressed (represented by the blue blanket) as HSCs transition to a largely quiescent population. The loss of this suppression of proliferation can result in the aberrant accumulation of immature HSC-like cells (e.g. in leukaemia), but may also be harnessed to expand HSCs transiently outside the body for clinical utility.

One of the key distinguishing properties between FL and adult HSCs is their cell cycle status: FL HSCs are actively cycling whereas adults HSCs are mostly quiescent. Therefore, another important developmental transition is the switch between these two states (Fig. 1). Specifically, in a process initiated before birth, and lasting until approximately 3-4 weeks postpartum in mice, a gradual shift in the cellular and molecular properties of HSC occurs (Bowie et al., 2007a,b, Copley and Eaves, 2013; Kim et al., 2007; Li et al., 2020). Multiple foetal-specific characteristics are lost and an adult HSC phenotype is established. This includes entering a state of quiescence with reduced rates of translation (Kohli and Passegué, 2014), a shift from oxidative phosphorylation to anaerobic, glycolytic-based metabolism (Kohli and Passegué, 2014; Manesia et al., 2015; Yu et al., 2013), a shift in the expression of key HSC regulators (Bowie et al., 2007a,b, Kim et al., 2007; Copley et al., 2013; Jassinskaja et al., 2017) and a re-balancing of mature cell outputs (Signer et al., 2014). This is further accompanied by a switch from the production of an innate-like lymphoid compartment (Ikuta et al., 1990; Hardy and Hayakawa, 1991) towards the production of adaptive T and B cells (Gilfillan et al., 1993) and, during the final stages of the foetal-to-adult switch at 3-4 weeks, bone mineralisation significantly increases, perhaps indicative of a larger organismal transition period (Ferguson et al., 2003). Interestingly, there is also a metabolic switch that developing HSCs undergo and this is additionally associated with a decrease in reactive oxygen species (ROS) levels (Pimkova et al., 2022), and maintenance of a ROS^low^ state is essential for adult HSC function (Ito et al., 2004; Ito et al., 2006). Also of note, as adult HSCs initiate differentiation, they undergo a further metabolic switch back to oxidative phosphorylation, which becomes the primary energy source for downstream progenitors, enabling a larger energy output.

## Key differences between foetal and adult HSCs

As a result of the foetal-to-adult switch, several differences between FL and adult HSCs are evident, especially with regard to their expansion rates and developmental potential. During development, FL HSCs undergo massive expansion, increasing in numbers by 10-30 fold within 4 days (Ema and Nakauchi, 2000). Although there is evidence of some seeding of HSCs generated from, and/or expanded in, the placenta (Rhodes et al., 2008; Ottersbach and Dzierzak, 2005), most of the increase in FL HSCs is due to their frequent execution of symmetrical self-renewal divisions (Bowie et al., 2006; Morrison et al., 1995). This higher rate of expansion was foreshadowed by early experiments using FL HSCs, where it was shown that spleen colony-forming units (CFU-S) were a more rapidly regenerating stem/progenitor population compared with adult spleen and bone marrow HSCs (Becker et al., 1965).

FL HSCs also exhibit faster cycling rates compared with adult HSCs; nearly all FL HSCs are cycling whereas >75% of adult HSCs are quiescent (Cheshier et al., 1999). Despite this, the cell cycle transit time of FL HSCs is similar to that of adult HSCs once the latter have fully exited quiescence (Bowie et al., 2007a,b). The switch in cycling is an intrinsically regulated process, with the site change from the FL to the developing BM not inducing HSC quiescence until 3-4 weeks later (Bowie et al., 2007a,b).

FL HSCs possess a similar developmental potential to adult HSCs with respect to the types of cells that they can give rise to in bone marrow transplantation experiments (Bowie et al., 2007a,b), with the exception of some distinct developmental T-cell lineages (Ikuta et al., 1990). However, their stepwise progression through progenitor stages is much less well defined. At the population level, FL HSCs are more likely to be balanced in their mature cell output in functional assays (e.g. they contribute equally to myeloid and lymphoid lineages) compared with adult HSCs, which become more lymphoid deficient with age (Benz et al., 2012). Relative to adult HSCs, FL HSCs additionally show an erythroid-over-myeloid bias in both mice and humans (Rowe et al., 2016a,b, Popescu et al., 2019). Recent work has also shown a developmental switch in the megakaryocyte production capacity of HSCs, with foetal HSCs being considerably less primed towards the megakaryocytic lineage compared with adult HSCs (Kristiansen et al., 2022). Using the HSC subtypes described by Dykstra et al. (2007), each cell can be categorised based on its mature cell production in single-cell transplantation assays as alpha, beta, gamma or delta. Interestingly, in line with the 3-4 week switch, ratios of the HSC subtypes present at this timepoint shift, suggesting a wider change in HSC functionality (Benz et al., 2012, Copley et al., 2013).

In functional assays, in particular during the early stages following transplantation, FL HSCs repopulate recipients more quickly than their adult counterparts and generate more HSCs through symmetric self-renewal (Bowie et al., 2007a,b, Pawliuk et al., 1996; Micklem et al., 1972). Around 6 weeks after transplantation, FL HSCs adopt a more adult-like self-renewal capacity, resembling the natural transition that occurs between 3 and 4 weeks after birth (Bowie et al., 2007a,b).

## Molecular regulation of the foetal-to-adult transition in HSCs

Two cardinal properties of HSCs are valuable to consider from a molecular standpoint when trying to understand HSC expansion and cancer development. First, what is the molecular state of an FL HSC undergoing self-renewal expansion division? This is key to understanding HSC expansion. Second, how are HSCs programmed to ‘go to sleep’ during the 3-4-week period, and how then are they ‘awoken’ during cancer development?

Recent work has shown that, although most molecular changes in developing HSCs are uncoordinated on a single-cell level, nearly all HSCs show a transient spike in type I interferon (IFN) signalling and expression of associated genes between E16.5 and postnatal day (P) 0 (Li and Magee, 2021). These findings correspond with previous work showing that differential expression of type I IFN-associated transcripts and proteins is one of the key molecular differences between FL and adult HSCs (McKinney-Freeman et al., 2012; Kim et al., 2016; Jassinskaja et al., 2017), and other studies identifying type I IFN signalling as a driver of AGM HSC generation and maturation (Kim et al., 2016; Li et al., 2014).

Several genes have also been linked directly to the regulation of FL or adult HSCs. One major complex is the Lin28b-let7-Hmga2 axis, which has been shown to be a key pathway, and master regulator, of the FL state (Copley et al., 2013; Rowe et al., 2016a,b). In FL HSCs, *Lin28b* and *Igf2bp3* maintain the foetal HSC phenotype by forming a complex that stabilises the expression of key FL HSC genes (including *Hmga2* and *Arid3a*). Importantly, both *Lin28b* and *Igfb3* are downregulated postnatally and so are effectively foetal specific. Inducing ectopic expression of *Lin28b and Igf2bp3* is enough to revert adult HSCs to a phenotypically foetal state, and this is accompanied by an associated increase in proliferation and erythroid bias. These induced foetal-like cells are also able to generate foetal-specific IL-7RA-expressing LMPPs and have an increased capacity to generate innate-like B-cell subsets (Rowe et al., 2016a,b; Wang et al., 2019; Kristiansen et al., 2016).

A number of other genes have been identified to be exclusively important for embryonic HSCs, such as *Runx1* (Okuda et al., 1996; Chen et al., 2009; Wilson et al., 2011; Wilkinson and Gottgens, 2013) and *Ezh2* (Mochizuki-Kashio et al., 2011), or vice versa for adult HSCs, such as *Bmi1* (Park et al., 2003), *Gfi1* (Wilson et al., 2010) and *Cebpa* (Ye et al., 2013). Perhaps the most comprehensive study of the foetal-to-adult transition revealed *Sox17* as a foetal and neonatal HSC-specific gene, with little to no expression detected in adult HSCs (Kim et al., 2007). Here, an induced germline deletion of *Sox17* resulted in the lack of any detectable HSCs, whereas conditional deletion of *Sox17* from the haematopoietic cell compartment using a *Tie2-Cre* floxed allele of *Sox17* (*Tie2-Cre^+^Sox17^fl/GFP^*) resulted in the loss of foetal and neonatal, but not adult HSCs (Kim et al., 2007). It is possible, however, that other Cres may not give the same result. Importantly, the reduction of *Sox17* expression aligns with the 3-4 week developmental switch and is consequently linked to reduced self-renewal and proliferation, and the acquisition of the adult HSC phenotype.

Transcription factors such as Sox17 are not the only regulators that differ across FL and adult HSCs. The cytokine stem cell factor (SCF; KITL) and its receptor Kit play major roles in regulating HSC numbers (Metcalf, 2008; McCulloch et al., 1965) and in the microenvironment-mediated control of HSCs (McCulloch et al., 1964). Although FL HSCs are strongly dependent on Kit activation, they require much less SCF than do adult HSCs despite similar levels of surface Kit expression (Bowie et al., 2007a,b). Thrombopoietin (TPO) also plays a role in HSC self-renewal (Audet et al., 2001). Indeed, knockout studies show that TPO-null mice have decreased numbers of repopulating HSCs (Solar et al., 1998) and that genetic deletion of *Mpl*, the TPO receptor, reduces HSC self-renewal potential (De Graaf and Metcalf, 2011). Additionally, genetic perturbation of LNK (SH2B3), a negative regulator of TPO signalling expression of which increases with age, increases HSC self-renewal (Seita et al., 2007). LNK acts by negatively regulating JAK2, a receptor tyrosine kinase downstream of many different cytokines, including TPO, IL6 and IL11 (Gery et al., 2009). A single LNK-deficient HSC can expand approximately 3000-fold after transplantation (Ema et al., 2005). Notably, TPO and *Mpl* are dispensable for FL HSC survival and expansion (Qian et al., 2007).

Experimentally, cytokines have many advantages for use in HSC expansion and have been combined in a wide range of multi-factorial screening studies. Their ease of use, the reversibility of their effects by removal, and the lack of permanent DNA manipulations make cytokines very attractive for stimulating HSC self-renewal. An early study optimised and assessed the effects of four key cytokines linked to HSC *in vitro* expansion (TPO, FLT3 ligand, SCF and IL11), performing an extensive two-level factorial analysis, testing every possible factor combination at two different concentrations, respectively (Audet et al., 2002). SCF and IL11 were found to be the most potent stimulators of HSC expansion. Based on this and a study by Miller and Eaves (1997), it was concluded that TPO offers no beneficial effect to adult HSC expansion and this was later extended to FL HSCs (Bowie et al., 2007a,b). However, a number of other groups have offered a different perspective, suggesting that TPO is crucial for HSC *in vitro* self-renewal (Nakauchi et al., 2001; Kimura et al., 1998; Solar et al., 1998; Wilkinson et al., 2020; Chou and Lodish, 2010). Explanations for these differences could include the effects of differing base medium and various supplements. Regardless, a large number and variety of cytokine conditions have been optimised over the years, largely still focused on TPO, SCF and gp130 (IL6ST) stimulants (e.g. IL6, IL11).

## Translating insights from development to the clinic

Differences in the intrinsic gene regulatory networks and extrinsic cues that regulate FL and adult HSCs ensure that the demands of each ontological stage are met. For FL HSCs, this involves the rapid expansion and establishment of the haematopoietic system, whereas in the adult it is HSC regulation and homeostasis. By harnessing knowledge of these regulatory pathways and signals, we can work towards recapitulating the appropriate environment *in vitro* to improve HSC expansion. In addition to allowing large-scale screens, such as proteomics and metabolomics, to be undertaken with sufficient cell numbers, improved HSC expansion would unlock a wide clinical benefit, with implications for BM rescue, gene therapy and a better understanding of childhood and adult leukaemias.

Recently, exciting progress has been made in the development of polyvinyl alcohol (PVA)-based HSC expansion cultures and these have been revolutionary for the field, allowing up to 899-fold increases in HSC numbers over a 28-day culture period (Wilkinson et al., 2019). Although these cultures are reductionist and intentionally synthetic, in order to avoid batch variability in reagents such as foetal bovine serum, they are grounded on the information gained over the last decades on SCF and TPO concentrations during the FL expansion phase. In PVA cultures (in contrast to the adult BM, where much higher cytokine doses are observed), low SCF (10 ng/ml) is combined with high TPO (100 ng/ml) to achieve the best expansion results (Wilkinson et al., 2019; Bowie et al., 2007a,b).

Interestingly, studies using this PVA protocol show that expansion of the phenotypic HSC compartment occurs largely independently of total cell proliferation in the culture and, as a result, overall cell number is a poor surrogate for HSC expansion (Wilkinson et al., 2019; Che et al., 2022). This is in accordance with previous studies showing that cytokine conditions stimulating rapid proliferation and higher cell numbers often do not yield the most functional HSCs (Audet et al., 1998). Prior to the Wilkinson et al. study, the best expansion attempts with haematopoietic cytokines only achieved maintenance for a week or two at most (Yamazaki and Nakauchi, 2014), suggesting that cytokines alone are limited in their capacity to maintain long-term self-renewal. As a result, numerous other strategies, such as transgene expression, soluble factors and supportive co-cultures, were developed in attempt to expand HSCs *ex vivo* (Sekulovic et al., 2011; Ohta et al., 2007). A recent example involving transgenic overexpression of *Mir130b* and *Mir128a* (*Mir128-1*), which are upregulated in the highly proliferative leukaemic stem cells underpinning childhood leukaemias, suggests that HSC expansion might be improved by forcing HSCs to expand more rapidly than normal (Malouf et al., 2021). Furthermore, the addition of bile acids, which protect rapidly expanding FL HSCs against endoplasmic reticulum stress *in utero* (Sigurdsson et al., 2016), to cell culture media was recently shown to improve the *ex vivo* expansion of adult HSCs (Koide et al., 2022).

Notably, the studies discussed above focus on mouse HSCs, as efforts to expand human HSCs have not been so successful. Historically, expansion of human HSCs *in vitro* has proved significantly more challenging than that of mouse with only modest increases in the number of transplantable HSCs being achieved. Several studies involving cytokines alone achieved a 3-fold increase in HSC number after 10 days using serum-free media supplemented with various combinations of IL6, IL3, IL11, FLT3 ligand, granulocyte colony-stimulating factor, and SCF (Miller and Eaves, 1997; Bhatia et al., 1997). More recently, groups have succeeded in gaining 3- to 20-fold increases in CD34^+^ human HSC numbers using PVA-based media after 7 (Wilkinson et al., 2019) or 14 (Sudo et al., 2021) days. This modest expansion prompted a number of groups to then explore the addition of small molecules such as StemRegenin 1 (SR1) (Boitano et al., 2010) and UM171 (Fares et al., 2014), both of which substantially improve the *in vitro* expansion of human HSCs. UM171 elicits its effects via activation of the ubiquitin ligase cullin 3 (CUL3) and subsequent polyubiquitylation and degradation of the LSD1-CoREST epigenetic regulating complex. This ensures the maintenance of H3K4me2 and H3K27ac marks, which are typically lost rapidly in human HSCs in *in vitro* culture (Fares et al., 2014). SR1 antagonises the aryl hydrocarbon receptor and selectively promotes the expansion of human CD34^+^ cells 12- to 17-fold, while also inhibiting proliferation of the CD34^−^ population (Boitano et al., 2010). Collectively, however, the limited number of long-term serially transplantable HSCs and their purity in expansion cultures remain major obstacles for the field.

## Emerging areas: mechanical biology and bioengineering

In order to mimic the supportive properties of the HSC niche, several studies over the last decades have utilised the co-culture of HSCs with various feeder cells derived from different sources that naturally support HSC expansion. These include cells from the AGM (Morrison and Scadden, 2014), urogenital ridge (Oostendorp et al., 2002), FL (Buckley et al., 2011) and BM (Zhang and Lodish, 2004). In particular, the AGM-S3, AFT024, UG26-1B6 (UG26) and EL08-1D2 (EL08) cell lines have been shown to support the survival and maintenance of adult mouse HSCs for at least 6 weeks in culture (Moore et al., 1997; Oostendorp et al., 2002). Notably, Oostendorp et al. demonstrated that the supportive effect of UG26 and EL08 cells does not necessarily require direct cell-to-cell contact with HSCs, suggesting that secreted factors are sufficient (Oostendorp et al., 2002; Xu et al., 1998). Several studies have additionally suggested that mesenchymal stem and progenitor cells can support HSC activity in co-cultures (Méndez-Ferrer et al., 2010; De Lima et al., 2012).

With respect to specific components of the HSC BM niche that regulate the maintenance and differentiation of HSCs, there have been numerous studies that have interrogated the cell types and molecular signals at play. Paradoxically, almost every cellular constituent of the BM has been suggested to play a role in HSC biology (Morrison and Scadden, 2014; Pinho and Frenette, 2019; Boulais and Frenette, 2015), with some groups hypothesising that distinct niches exist for different HSC subpopulations (Pinho et al., 2018). This latter idea is further complicated by the fact that HSCs are a heterogeneous population with distinct properties, and no HSC reporter yet exists with 100% specificity for HSCs or specific HSC subtypes, making definitive investigations challenging to conduct.

The mechanical properties of the HSC niche also appear to be crucial during development for both the maintenance and regulation of established foetal and adult HSC populations, for haematopoietic commitment and development, and for the initial generation of HSCs via EHT. Given the important role played by the HSC niche, it is conceivable that the historic lack of success in HSC *ex vivo* expansion is at least in part due to the inability of liquid cultures or even stromal co-cultures to satisfy the three-dimensional and mechanical aspects of the HSC niche. Consequently, bioengineering approaches that allow us to both understand the biological importance of, and more accurately imitate, the HSC niche *in vitro* are highly relevant and hold great potential. This is an ongoing challenge, facilitated through the engineering of artificial 3D niches with ECM proteins and functionalised hydrogels (Bai et al., 2019). In order to mimic the niche accurately, matrix stiffness and ligand type and spatial distribution are important factors that must be considered. Stiffness of the niche has already been linked to HSC morphology, mobility and cell adhesion, and ligand type has a significant impact on the lineage biases of HSCs (Li et al., 2021).

Recently, optical scaffolds made of 3D nanofibers have been demonstrated to permit the culture of cells on structures that maintain high porosity for cell migration and nutrient transport and that are perhaps more realistic models of HSC growth *in vitro*. Nanofibers may be biological (collagen/fibrin/tropoelastin) or synthetic [polycaprolactone (PCL), polyethylene terephthalate (PET), polyurethane (PU), ceramics], a hybrid of both, and/or functionalised with molecules such as CXCL12 to improve HSC culture (Li et al., 2021). Ceramic electrospun nanofibers are particularly exciting and relevant to the study of HSCs, having been shown to mimic a number of bone properties (Esfahani et al., 2017). The diameter of these nanofibers as well as their density and pore size influence the behaviour of cells that are cultured upon them. One example used umbilical cord HSCs, showing that HSCs expand 178- to 194-fold in these 3D cultures compared with 50-fold when using traditional 2D plates (Chua et al., 2006), and another study achieved ∼550-fold increases in CD34^+^ cell numbers (Das et al., 2009). Moreover, murine embryonic stem cells have been shown to have increased survival, proliferation and phenotypic HSC-specific differentiation when cultured in 3D artificial niches (Dehdilani et al., 2016). Finally, MS-5 stromal cells have been used to produce extracellular matrices *in vitro*, which can then act as scaffolds for culturing CD34^+^ human cord blood cells (Tiwari et al., 2013) and it was shown that the acellular scaffolds increase phenotypic HSCs and CFUs by 80-fold.

Microfluidics can also be used to manipulate and control liquids in small volumes (of 10^−9^ to 10^−18^ litres) using channels that are tens of microns in diameter. These are proving to be incredibly useful platforms, especially for mimicking cardiovascular forces, such as sheer stress, and can also be scaled up for use in bioreactors (Islam et al., 2017; Whitesides, 2006). The future application of such microfluidic approaches in the context of HSC expansion could also provide exciting results.

## Future perspectives

We have witnessed an enormous amount of recent activity and progress in the field of HSC expansion, and, for the first time, functional murine HSCs can be expanded *ex vivo* robustly and durably. There is therefore a huge opportunity to re-imagine experiments that were previously thought to be impossible owing to cell number issues. The transcriptomes of actively expanding HSCs in these cultures have been shown to be highly similar to those of native FL HSCs (Che et al., 2022), with small differences possibly explained by the absence of an HSC niche or appropriate metabolic conditions. Indeed, a key characteristic of the 3-4 week switch is the metabolic change from aerobic oxidative phosphorylation in foetal HSCs, to anaerobic glycolysis in adult HSCs (Kohli and Passegué, 2014). This accompanies the change from an actively cycling to a largely quiescent population and highlights the possibility of metabolic manipulation in order to recover a more active, aerobically metabolising, cellular state. This could have substantial clinical applications, as it is known that leukaemic stem cells have a greater dependence on aerobic, mitochondrial respiration than the normal adult HSC population (De Beauchamp et al., 2022). A blanket inhibition of oxidative phosphorylation might therefore allow healthy HSCs to survive via adaptation to glycolysis-mediated metabolism, while killing leukaemic stem cells.

Larger-scale experiments, potentially in combination with hydrogels and/or mechanical stresses, are also now possible and could be used to identify the key pathways that govern HSC expansion and translate these findings to human HSC biology. Fed-batch systems, which provide an automated and continuous supply of fresh media to the cultures, are already being applied with novel small molecules such as UM171 and SR1 (Fares et al., 2014) to achieve improved levels of expansion. Combining such promising avenues will undoubtedly lead to success in future clinical-scale human HSC expansion.
